# Angiotensin II Inhibits Insulin Receptor Signaling in Adipose Cells

**DOI:** 10.3390/ijms23116048

**Published:** 2022-05-27

**Authors:** Citlaly Gutierrez-Rodelo, Araceli Arellano-Plancarte, Judith Hernandez-Aranda, Huguet V. Landa-Galvan, G. Karina Parra-Mercado, Nicole J. Moreno-Licona, Karla D. Hernandez-Gonzalez, Kevin J. Catt, Rafael Villalobos-Molina, J. Alberto Olivares-Reyes

**Affiliations:** 1 Laboratory of Signal Transduction, Department of Biochemistry, Center for Research and Advanced Studies of the National Polytechnic Institute, Cinvestav-IPN, Mexico City 07360, Mexico; citlalygutierrezrodelo@gmail.com (C.G.-R.); araplan@yahoo.com (A.A.-P.); juhernadez@cinvestav.mx (J.H.-A.); hlanda@cinvestav.mx (H.V.L.-G.); gparra@cinvestav.mx (G.K.P.-M.); nicole.moreno@cinvestav.mx (N.J.M.-L.); daniela.hernandez@cinvestav.mx (K.D.H.-G.); 2 Section on Hormonal Regulation, PDEGEN, National Institute of Child Health and Human Development, NIH, Bethesda, MD 20892, USA; cattk@mail.nih.gov; 3 Biomedicine Research Unit, Faculty of Higher Studies, FES-Iztacala, National Autonomous University of Mexico, UNAM, Edo. Mex., Tlalnepantla 54090, Mexico; villalobos@unam.mx

**Keywords:** adipose cells, angiotensin II, insulin receptor, insulin resistance, protein kinase C, serine-phosphorylation

## Abstract

Angiotensin II (Ang II) is a critical regulator of insulin signaling in the cardiovascular system and metabolic tissues. However, in adipose cells, the regulatory role of Ang II on insulin actions remains to be elucidated. The effect of Ang II on insulin-induced insulin receptor (IR) phosphorylation, Akt activation, and glucose uptake was examined in 3T3-L1 adipocytes. In these cells, Ang II specifically inhibited insulin-stimulated IR and insulin receptor substrate-1 (IRS-1) tyrosine-phosphorylation, Akt activation, and glucose uptake in a time-dependent manner. These inhibitory actions were associated with increased phosphorylation of the IR at serine residues. Interestingly, Ang II-induced serine-phosphorylation of IRS was not detected, suggesting that Ang II-induced desensitization begins from IR regulation itself. PKC inhibition by BIM I restored the inhibitory effect of Ang II on insulin actions. We also found that Ang II promoted activation of several PKC isoforms, including PKCα/βI/βII/δ, and its association with the IR, particularly PKCβII, showed the highest interaction. Finally, we also found a similar regulatory effect of Ang II in isolated adipocytes, where insulin-induced Akt phosphorylation was inhibited by Ang II, an effect that was prevented by PKC inhibitors. These results suggest that Ang II may lead to insulin resistance through PKC activation in adipocytes.

## 1. Introduction

Insulin resistance is a systemic condition characterized by impaired insulin signaling and glucose uptake. At the molecular level, dysregulation of the insulin receptor (IR) and insulin receptor substrate (IRS) proteins is a common feature of insulin resistance [1,2,3]. Some mechanisms that could explain how this dysregulation occurs include: (1) A decreased number of IRs and their tyrosine kinase activity; (2) increased serine/threonine (Ser/Thr) phosphorylation of both proteins; and (3) increased activity of protein tyrosine phosphatases (PTPs), which participate in dephosphorylation and regulation of IR and IRS [2,4]. Phosphorylation of Ser/Thr-residues of IR and IRS-1 proteins constitutes an essential factor contributing to the development of insulin resistance [5,6]. It has been demonstrated that IR/IRS-1-Ser/Thr-hyperphosphorylation decreases their Tyr-phosphorylation and reduces insulin receptor kinase activity and IRS-1 interactions with downstream substrates such as PI3K, altering phosphorylation and activation of Akt kinase [7,8]. Protein kinases such as MAPK, JNK, PKA, and PKC have been identified as potential Ser/Thr kinases associated with phosphorylation and IR regulation [3,5]. In particular, it has been demonstrated that an incremented PKC activity is associated with insulin resistance states in several cell models and humans [2,9].

One of the well-characterized inductors of insulin resistance is angiotensin II (Ang II), the main bioactive peptide of the renin-angiotensin-system (RAS) [4]. Ang II plays an essential role in cardiovascular and renal systems, and alterations in its actions have been associated with different pathologies, including endothelial dysfunction, atherosclerosis, myocardial infarction, congestive heart failure, hypertension, and renal disease [10]. However, additional studies have shown that Ang II may induce insulin resistance, whereas Ang II receptor blockers and the angiotensin-converter-enzyme inhibitors enhance insulin sensitivity [11].

The best-characterized actions of Ang II on insulin signaling have been reported in the cardiovascular system [12,13] and insulin-sensitive tissues such as the liver [14,15] and skeletal muscle [11,16]. In these tissues, Ang II affects the activation and function of IR, IRS, and the downstream effectors phosphatidylinositol 3-kinase (PI3K), Akt, and the glucose transporter 4 (GLUT4) [4]. Through the angiotensin receptor type 1 (AT_1_R) activation, Ang II promotes Ser/Thr-phosphorylation of IRS on different sites mainly via reactive oxygen species (ROS) generation, Ser/Thr kinases activation, and the epidermal growth factor receptor transactivation [4,14,16]. Additionally, Ang II impairs the Tyr-phosphorylation of IR by activation of PTPs and through the synthesis of suppressor of cytokine signaling-3 (SOCS-3), which reduces IR/IRS interaction [17,18].

In contrast, in human adipocytes and 3T3-L1 adipose cells, it has been reported that Ang II inhibits lipolysis and promotes lipogenesis [19,20,21], which are opposite to its reported catabolic actions [22,23,24] and similar to the insulin effects in this tissue [25]. Diverse investigations have been directed to clarify the association of Ang II with insulin resistance-linked obesity [26,27]. For example, there is evidence indicating that under obesity, the local adipose RAS tissue, particularly white visceral adipose tissue (VAT), is increased in humans and animals and clearly correlates with elevated local and systemic Ang II levels, which promotes hypertrophic adipose tissue because of lipid accumulation, adipocyte growth, and differentiation which in turn secrete adipokines [28]. Therefore, Ang II drives hypertension, dyslipidemia, and insulin resistance, fueling obesity [29]. In turn, the high adipose mass leads to further perturbation in blood pressure, lipid, and glucose level. Hence, obesity carries the risk of type 2 diabetes mellitus (DM2) and cardiovascular and kidney diseases, triggering the vicious cycle of these pathologies [29,30]. Although there is a clear relationship between Ang II and its role in developing insulin resistance in the cardiovascular, hepatic, and muscular systems [4], the effect of Ang II on insulin signaling in adipose cells, it is still under discussion.

While some studies have shown that Ang II does not promote any alteration at the insulin receptor binding level or activity [31,32], others have proposed that chronic Ang II infusion potentiates insulin sensitivity in isolated rat adipocytes. However, Ang II infusion decreases insulin secretion [33] and induces systemic insulin resistance [34]. There is also evidence that at the molecular level, Ang II impairs insulin-induced Akt activation and its downstream substrates glycogen synthase kinase-3 (GSK-3β) and AS160 in isolated rat adipocytes [35]. Furthermore, it has been observed that Ang II diminishes glucose uptake by decreasing GLUT4 translocation to the membrane under insulin stimulus in isolated adipocytes [34]. Ang II can also enhance SOCS-3 expression, which has been related to insulin signaling attenuation in 3T3-L1 adipocytes [36]. However, despite the existing evidence on the effect of Ang II on insulin signaling in adipose cells, the exact molecular mechanisms associated with this effect remain under discussion. Thus, in the present study, it was decided to examine the effect of Ang II on insulin signaling and the potential molecular mechanisms associated with 3T3-L1 adipose cells. Here we show that Ang II activation of endogenous AT_1_R impairs insulin-stimulated Tyr-phosphorylation of the IR and IRS, reducing Akt activation, FoxO1 regulation, and glucose uptake. Interestingly, protein kinase C (PKC) inhibition restores the inhibitory effect of Ang II on insulin-stimulated Tyr^1158^-phosphorylation of the IR, Akt phosphorylation at Ser^473^, and glucose uptake. These results suggest that Ang II may lead to insulin resistance through PKC activation in 3T3-L1 adipocytes.

## 2. Results

### 2.1. Effect of Ang II on Insulin-Induced Signaling in 3T3-L1 Adipocytes

**Figure 1:**
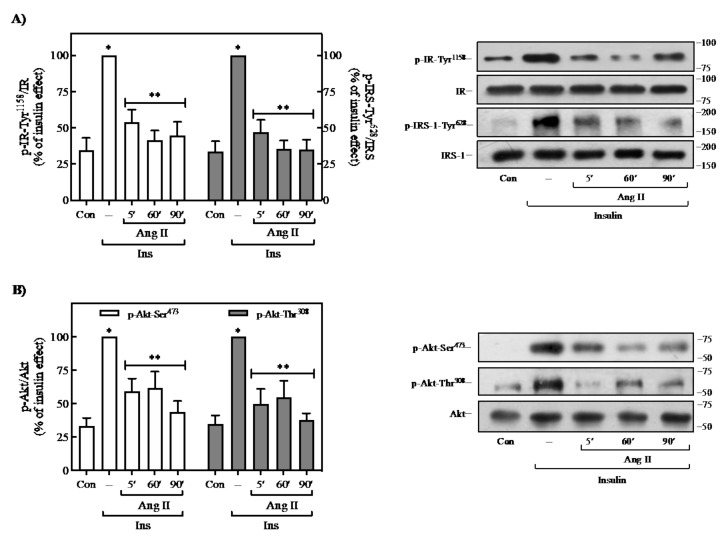
Effect of Ang II on insulin-induced IR, IRS, and Akt phosphorylation. (**A**,**B**) 3T3-L1 adipocytes were pre-treated with 100 nM Ang II from 5 to 90 min and then stimulated with 100 nM insulin for an additional 90 min. Under all experimental conditions, cells without treatment were considered as controls. Total cell lysates were separated by SD-PAGE and analyzed by immunoblotting with anti-p-IR Tyr^1158^, anti-p-IRS Tyr^628^ (**A**), anti-p-Akt-Ser^473^, or an-ti-p-Akt-Ser^308^ (**B**), as described in Methods. IR, IRS-1, and Akt phosphorylation was quantitated by densitometry, and the mean values were plotted from three–five independent experiments. Vertical lines represent the S.E.M. Representative immunoblots are presented. Western blots were also probed for total IR and IRS (**A**), and Akt (**B**) and used for data normalization. (**A**,**B**) * *p* < 0.05 vs. Con; ** *p* < 0.05 vs. Ins (--). Con, control; Ins, insulin.

In recent years, several reports have shown that Ang II is an essential regulator of insulin signaling in metabolic tissues and the cardiovascular system. However, in adipose cells, the regulatory role of Ang II on insulin actions is still under debate. To investigate whether Ang II impairs insulin signaling, differentiated 3T3-L1 adipocytes were stimulated with 100 nM Ang II for 5, 60, or 90 min, followed by the addition of 100 nM insulin for 90 min. In 3T3-L1 adipocytes, where insulin exerts a clear effect on IR/IRS-1/Akt signaling pathway activation (Supplementary Figure S1), pretreatment with Ang II drastically reduced insulin-induced Tyr-phosphorylation of IR and IRS-1 in a time-dependent manner (Figure 1A). Similarly, and as expected, Ang II pretreatment caused a decrease in insulin-induced Akt phosphorylation at both Ser^473^ and Thr^308^ residues (Figure 1B). Thus, these results indicate that Ang II can desensitize the insulin-mediated signaling and its downstream effectors in adipose cells.

### 2.2. Effect of Ang II on Insulin-Induced Glucose Uptake and FoxO1 Regulation

**Figure 2:**
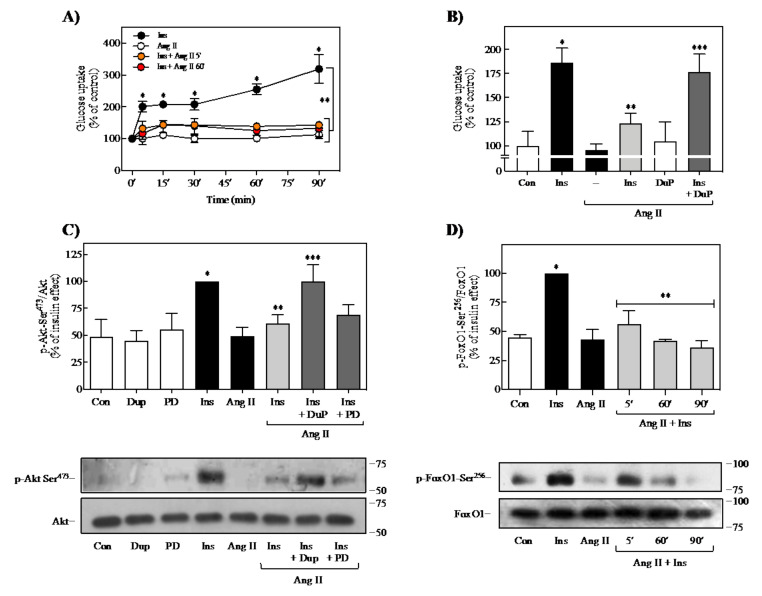
Effect of Ang II on insulin-induced glucose uptake and FoxO1 phosphorylation. (**A**) 3T3-L1 adipocytes were stimulated with 100 nM Ang II for the indicated times (white circles) or pretreated without (black circles) or with 100 nM Ang II for 5 (orange circles), or 60 min (red circles), and then stimulated with 100 nM insulin for the indicated times. (**B**) Cells were pretreated with 10 μM DuP 753 for 30 min before treatment with 100 nM Ang II for 60 min and then stimulated with 100 nM insulin for an additional 90 min. Glucose uptake was determined as described in Methods. (**C**) Cells were pretreated with or without 10 μM DuP 753 or 10 μM PD123177 for 30 min, then were stimulated with or without 100 nM Ang II for 60 min. Finally, the cells were stimulated with or without insulin for 90 min. (**D**) Adipocytes were pretreated with 100 nM Ang II for 5, 60, or 90 min, and then stimulated with 100 nM insulin for 90 min. Total cell lysates were separated by SDS-PAGE and analyzed by immunoblotting with anti-p-Akt Ser^473^ (**C**) and anti-p-FoxO1 Ser^256^ (**D**). Under all experimental conditions, cells without treatment were considered as controls. Blots were quantitated by densitometry, and the mean values were plotted from three-independent experiments. Vertical lines represent the S.E.M. Representative immunoblots are presented. Western blots were also probed to detect total Akt (**C**), and FoxO1 (**D**) and used for data normalization. (**A**) * *p* < 0.05 vs. time 0′; ** *p* < 0.05 vs. similar times stimulated only by Ins. (**B**,**C**) * *p* < 0.05 vs. Con; ** *p* < 0.05 vs. Ins; *** *p* < 0.05 vs. Ins + Ang II. (**D**) * *p* < 0.05 vs. Con; ** *p* < 0.05 vs. Ins. Con, control; DuP, DuP 753; PD, PD123177; Ins, insulin.

Subsequently, it was determined whether the observed effect of Ang II on insulin signaling is associated with impairment of insulin-induced glucose uptake in 3T3-L1 adipocytes. As shown in Figure 2A, insulin caused rapid and marked glucose uptake, reaching a maximum at 90 min of stimulation (~3.0-fold increase) (black circles). Ang II stimulation did not affect glucose uptake from 2 to 90 min (white circles). However, pretreatment with Ang II for 5 (orange circles) or 60 min (red circles) caused inhibition of insulin-induced glucose uptake (~80%) at all times of insulin treatment. This potent inhibition caused by Ang II was dependent on activation of the AT_1_R since pretreatment of cells with the highly specific AT_1_R antagonist losartan (DuP 753) reversed the effect (Figure 2B). These results suggest that Ang II pretreatment impairs insulin-induced glucose uptake.

To confirm that Ang II desensitizes insulin signaling through AT_1_R activation, we also evaluated the effect of DuP 753 on insulin-induced Akt phosphorylation. As mentioned earlier, Akt can phosphorylate target proteins that regulate glucose uptake and metabolism. As shown in Figure 2C, adipocytes treated with DuP 753 and further stimulated with Ang II and insulin showed a significant signal recovery effect. Although still controversial, previous studies have suggested that differentiated adipose cells also express AT_2_ receptors (AT_2_Rs) [37,38,39], even though the reported effects indicate that its activation counteracts those of AT_1_Rs [37,40]. However, in 3T3-L1 adipocytes, the use of a highly selective AT_2_R antagonist, PD123177, did not exert any change in Ang II regulation on insulin-induced phosphorylation of Akt (Figure 2C). These results suggest that the observed effect of Ang II exclusively depends on AT_1_R activation rather than the AT_2_R. Thus, together with Ang II inhibitory effect on insulin-induced IR-Tyr, IRS-Tyr, and Akt phosphorylation, the effects mentioned above could be involved in developing insulin resistance through AT_1_R activation. We also determined whether Ang II could affect the insulin-induced regulation of FoxO1, a transcriptional factor associated with lipolysis regulation in adipocytes [41]. As shown in Figure 2D, incubation of cells with 100 nM Ang II for 5, 60, or 90 min, followed by the addition of 100 nM insulin 90 min, also reduced insulin-induced FoxO1 phosphorylation on Ser^256^.

### 2.3. Effect of Ang II on IR- and IRS-1-Ser-Phosphorylation

**Figure 3:**
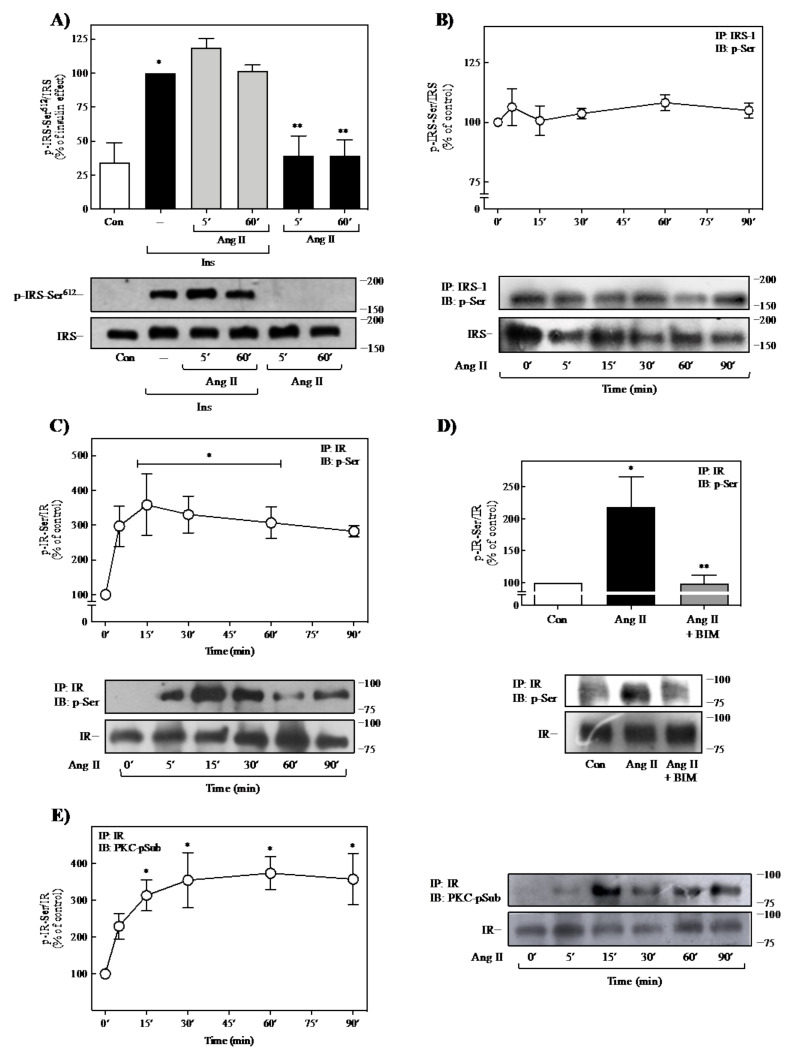
Ang II promotes IR-Ser-phosphorylation through PKC activation. (**A**) 3T3-L1 adipocytes were pretreated with or without Ang II for 5 or 60 min and then stimulated with 100 nM insulin for an additional 90 min. Cells were also exposed only to 100 nM Ang II for 5- or 60-min. Total cell lysates were separated by SDS-PAGE and analyzed by immunoblotting with anti-p-IRS-1-Ser^612^. (**B**,**C**,**E**) 3T3-L1 adipocytes were stimulated with 100 nM Ang II for the indicated times. Total cell lysates were immunoprecipitated with anti-IRS-1 (**B**) or anti-IR (**C**,**E**) antibody before SDS-PAGE analysis and immunoblotted with an anti-phospho-Ser antibody (**B**,**C**) or anti- phospho-(Ser) PKC substrate antibody (**E**). (**D**) Cells were pretreated with 2 μM BIM for 30 min before treatment with 100 nM Ang II for 60 min. Cell lysates were immunoprecipitated with anti-IR antibody before SDS-PAGE analysis and immunoblotted with an anti-phospho-Ser antibody. Under all experimental conditions, cells without treatment were considered as controls. Blots were quantitated by densitometry, and the mean values were plotted from three-five independent experiments. Vertical lines represent the S.E.M. Representative immunoblots are presented (**B**–**D**). Western blots were also probed for total IRS (**A**,**B**) and IR (**C**–**E**) and used for data normalization. (**A**) * *p* < 0.05 vs. Con; ** *p* < 0.05 vs. Ins (--). (**C**) * *p* < 0.05 and vs. time 0′. (**D**) * *p* < 0.05 vs. Con; ** *p* < 0.05 vs. Ang II. (**E**) * *p* < 0.05 vs. time 0. BIM, BIM I; Con, control; Ins, insulin.

There is evidence that increased phosphorylation of IR and IRS at Ser/Thr residues alters its autophosphorylation in response to insulin. Furthermore, phosphorylation at these residues on IR and IRS proteins reduces insulin signaling and may cause insulin resistance [3,42]. Previous studies have shown that phosphorylation of IRS at Ser^612^ residue represents a particular target of different Ser/Thr kinases, including PKC, and its phosphorylation has been associated with suppression of Akt activity [43,44]. Moreover, it has been identified that Ang II promotes phosphorylation of IRS at Ser^612^ as a negative regulatory mechanism of insulin signaling in human endothelial cells [45]. Thus, we first determined whether Ang II could induce IRS-1 phosphorylation at Ser^612^. As observed in Figure 3A, Ang II did not affect the insulin-induced IRS phosphorylation at Ser^612^. Moreover, Ang II alone could also not induce phosphorylation of IRS-1 at this specific residue. To define whether Ser-phosphorylation of IRS-1 or IR could be a potential mechanism of negative regulation by Ang II, we determined the effect of Ang II on the total phosphorylation state of Ser residues of both proteins. As shown in Figure 3B, the treatment with 100 nM Ang II was unable to promote total Ser-phosphorylation of IRS-1, compared with the effect of insulin (Supplementary Figure S2A).

In contrast, it was observed that 100 nM Ang II induced Ser-phosphorylation of IR in a time-dependent manner, reaching maximal activation at 5 min (~3.0-fold increase) and remaining until 90 min (Figure 3C). Similarly, we also found that insulin promotes this negative auto-regulatory mechanism in our study model (Supplementary Figure S2B). To determine whether PKC mediates the Ang II-induced Ser-phosphorylation of IR observed in Figure 3C, we evaluated the effect of the PKC inhibitor bisindolylmaleimide I (BIM I), a highly selective, cell-permeable, and reversible PKC inhibitor (Ki = 14 nM) [46]. As shown in Figure 3D, pretreatment of adipocytes with 1μM BIM I for 30 min prevented the effect of Ang II on the IR-Ser-phosphorylation. Additionally, we also determined the Ang II-induced phosphorylation of IR by PKC using a phospho-(Ser) PKC substrate antibody. Thus, we analyzed IR immunoprecipitates for the presence of phospho-(Ser) PKC substrate motifs. As shown in Figure 3E, Ang II caused phosphorylation of IR through PKC in a time-dependent manner, reaching a maximum at 15 min (a 3-fold increase over basal) and was sustained over the next 75 min. These results suggest that Ang II could regulate insulin signaling directly by modulating the IR but no IRS-1 in 3T3-L1 cells through phosphorylation of IR on Ser residues, a mechanism mediated by PKC. As confirmatory data, we also identified that only insulin, but not Ang II, induced the presence of phospho-(Ser) PKC substrate motifs in the IRS (Supplementary Figure S2C).

### 2.4. Role of PKC on Ang II-Mediated Regulation of Insulin Signaling

**Figure 4:**
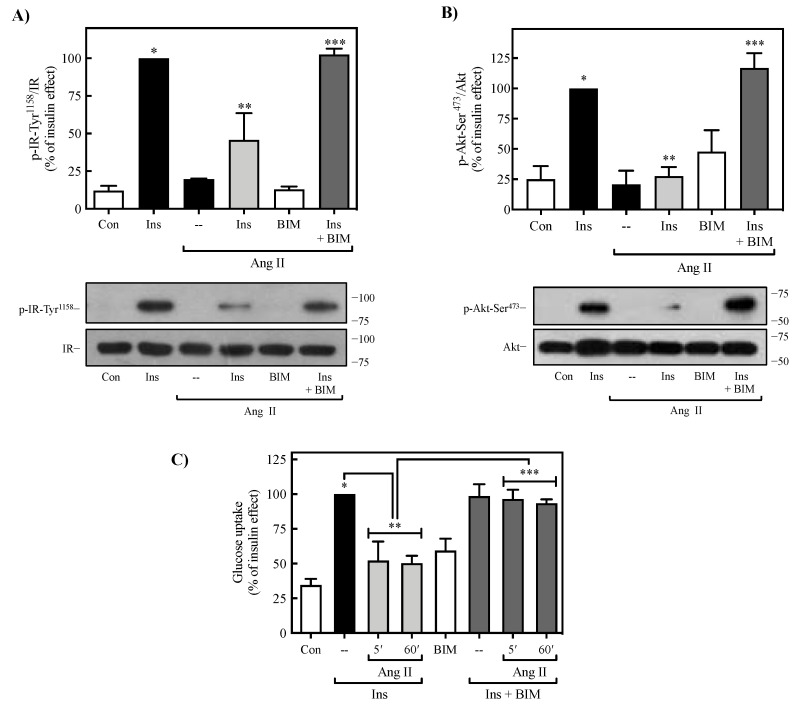
Role of PKC in Ang II-mediated regulation of insulin signaling. 3T3-L1 adipocytes were treated with or without 1 µM BIM for 30 min, then stimulated with or without 100 nM Ang II for 60 min. Finally, the cells were stimulated with or without insulin for 90 min. Total cell lysates were separated by SD-PAGE and analyzed by immunoblotting with anti-p-IR Tyr^1158^ (**A**) or anti-p-Akt Ser^473^ antibodies (**B**). Blots were quantitated by densitometry, and the mean values were plotted from three–five independent experiments. Western blots were also probed for total IR (**A**) and Akt (**B**) and used for data normalization. (**C**) Cells were treated with or without 1 μM BIM for 30 min, then stimulated with or without 100 nM Ang II for 5 or 60 min, and finally stimulated with or without insulin for 90 min. Glucose uptake was evaluated, as described in the Methods. Under all experimental conditions, cells without treatment were considered as controls. The values are reported as a percentage of insulin effect ± S.E.M. of at least three experiments. (**A**,**B**) * *p* < 0.05 vs. Con; ** *p* < 0.05 vs. Ins; *** *p* < 0.05 vs. Ins + Ang II. (**C**) * *p* < 0.05 vs. Con; ** *p* < 0.05 vs. Ins (--); *** *p* < 0.05 vs. Ang II + Ins (5′, 60′). BIM, BIM I; Con, control; Ins, insulin.

The canonical model indicates that the G_q_-coupled AT_1_R activation leads to inositol trisphosphate (IP3) and diacylglycerol production, causing intracellular Ca^2+^ mobilization and PKC activation; this signaling pathway plays essential roles in most of the biological and pathological actions of Ang II [4,10]. To determine whether PKC is involved in the observed inhibitory actions of Ang II on insulin-induced activation of IR, Akt, and glucose uptake, 3T3-L1 adipocytes were exposed to the PKC inhibitor BIM I (1 μM) for 30 min, treated with Ang II (100 nM for 5 or 60 min) and stimulated with insulin for 90 min. BIM I was found to inhibit the effect of Ang II on IR (Figure 4A) and Akt (Figure 4B) phosphorylation, indicating that its inhibitory effect on IR Tyr^1158^ and Akt Ser^473^ involves activation of PKC. Moreover, when the effect of BIM I was evaluated on the inhibitory action of Ang II on insulin-induced glucose uptake, this PKC inhibitor was able to completely reverse the effect of Ang II (Figure 4C). These findings indicate that Ang II-induced activation of PKC mediates its inhibitory effect on insulin signaling in 3T3-L1 adipocytes.

### 2.5. Ang II Promotes Activation of PKC Isoforms in 3T3-L1 Cells

**Figure 5:**
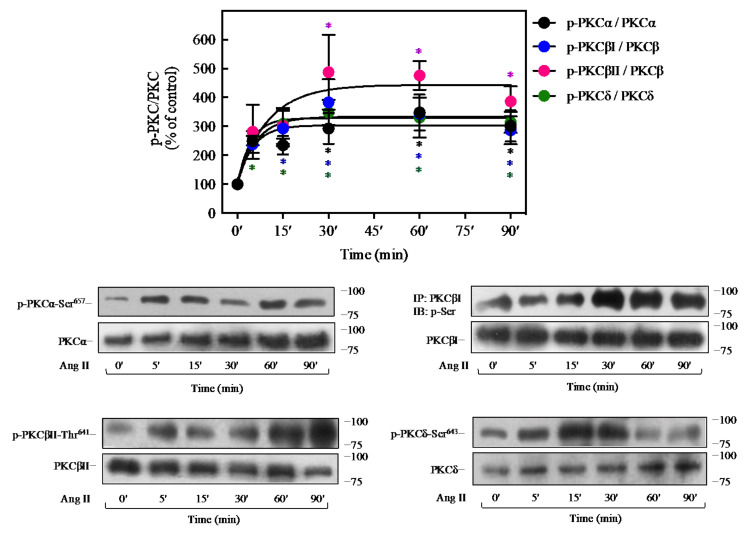
Ang II promotes the activation of PKC. 3T3-L1 adipocytes were treated with 100 nM Ang II for the indicated times. Under all experimental conditions, cells without treatment were considered as controls. Total cell lysates were separated by SD-PAGE and analyzed by immunoblotting with anti-p-PKCα Ser^657^, anti-p-PKCβII Thr^641^, or anti-p-PKCδ Ser^643^ antibodies. For PKCβI, total cell lysates were immunoprecipitated with anti-PKCβI before SDS-PAGE analysis and immunoblotted with an anti-phospho-Ser antibody. Blots were quantitated by densitometry, and the mean values were plotted from three-five independent experiments. Vertical lines represent the S.E.M. Representative immunoblots are presented. Western blots were also probed for total PKCα, PKCβI, PKCβII, or PKCδ as a loading control. * *p* < 0.05 vs. time 0′. The different asterisk colors indicate the corresponding group and represent the significance with respect to control within the same group.

Several studies on 3T3-L1 adipocytes have reported that these cells endogenously express members of the three PKC families: classical (PKCα, PKCβ), novel (PKCδ), and atypical (PKCλ, PKCζ) [47,48]. To investigate which isoforms of PKC are involved in the effect of Ang II on the insulin pathway, it was decided to evaluate the phosphorylation of PKC-isoforms that are expressed in these cells and are inhibited by BIM I, using a temporal course of Ang II from 5 to 90 min of stimulation. It was found by Western blot analysis that Ang II stimulus resulted in the phosphorylation of PKCα, PKCβI, and PKCδ in a time-dependent manner, reaching the maximal effect between 15 and 30 min (~3-fold increase) effect that was maintained up to 90 min of stimulation (Figure 5). Similarly, Ang II promoted PKCβII phosphorylation, reaching a maximal effect at 30 min of Ang II stimulation (about 4.5-fold increase) and decreasing at 90 min (~3.5-fold increase) (Figure 5, pink circles).

### 2.6. Ang II Induces the Interaction of PKC with IR

**Figure 6:**
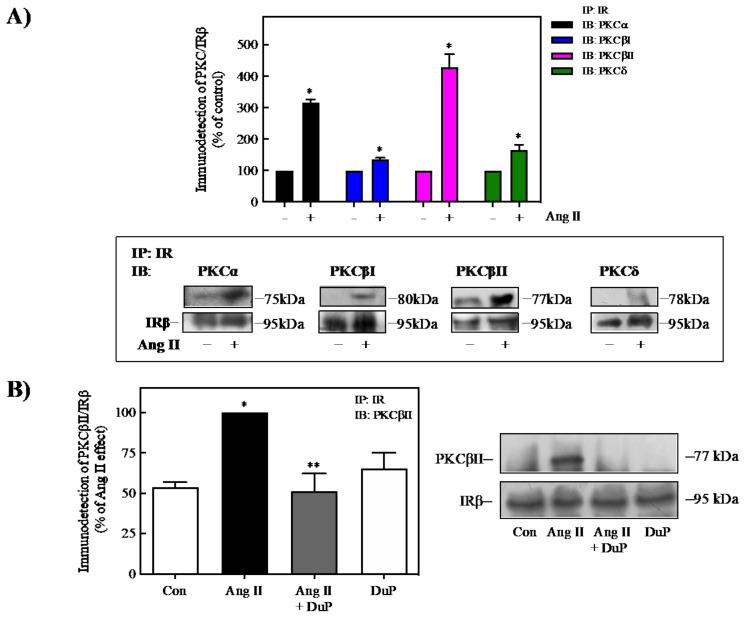
Ang II promotes the association of IR and classical and new isoforms of PKC. (**A**) 3T3-L1 adipocytes were pretreated with or without 100 nM Ang II for 60 min. (**B**) Cells were pretreated in the absence or presence of 10 μM DuP 753 for 30 min before treatment with 100 nM Ang II for 60 min. Under all experimental conditions, cells without treatment were considered as controls. Total cell lysates were immunoprecipitated with anti-IR before SDS-PAGE analysis and immunoblotted with anti-PKCα, anti-PKCβI, anti-PKCδ (**A**), or anti-PKCβII (**A**,**B**). Blots were quantitated by densitometry, and the mean values were plotted from three-five independent experiments. Vertical lines represent the S.E.M. Representative immunoblots are presented. Western blots were also probed for total IRβ. (**A**) * *p* < 0.05 vs. Con (−). (**B**) * *p* < 0.05 vs. Con; ** *p* < 0.05 vs. Ang II. Con, control; DuP, DuP 753.

Previous studies have identified that under stimulus that promotes insulin resistance, different isoforms of PKC, such as PKCα, PKCβI/II, and PKCδ, are associated with regulation of the IR-kinase activity, possibly through the interaction of PKC with the IR [49,50,51]. To investigate the role of PKC isoforms in the Ang II-mediated regulation of the IR, cell lysates from unstimulated and 100 nM Ang II-stimulated 3T3-L1 adipose cells (after 60 min stimulation) were immunoprecipitated with IR-antibody and blotted with selective anti-PKCα-, βI-, βII-, and δ-antibodies. As shown in Figure 6A, Ang II induced the association between the IR and PKCα, βI, βII, and δ compared with control cells. It was observed that Ang II induces a more significant association between the IR and PKCβII (~4.0-fold increase) compared with PKCα (~2.0-fold increase), PKCβI (~1.20-fold increase), or PKCδ (~1.5-fold increase). This association was prevented with the AT_1_R antagonist DuP 753 (Figure 6B). Thus, these data suggest that classical (α, βI, and βII) and new (δ) PKC isoforms are activated and associated with the IR to promote its Ser-phosphorylation under Ang II stimulation. Therefore, there is a decrease in IR kinase activity and impairment of Akt activation and glucose uptake in adipose 3T3-L1 cells.

### 2.7. Ang II Induces Insulin Resistance in Adipocytes Isolated from BALB/c Mice

**Figure 7:**
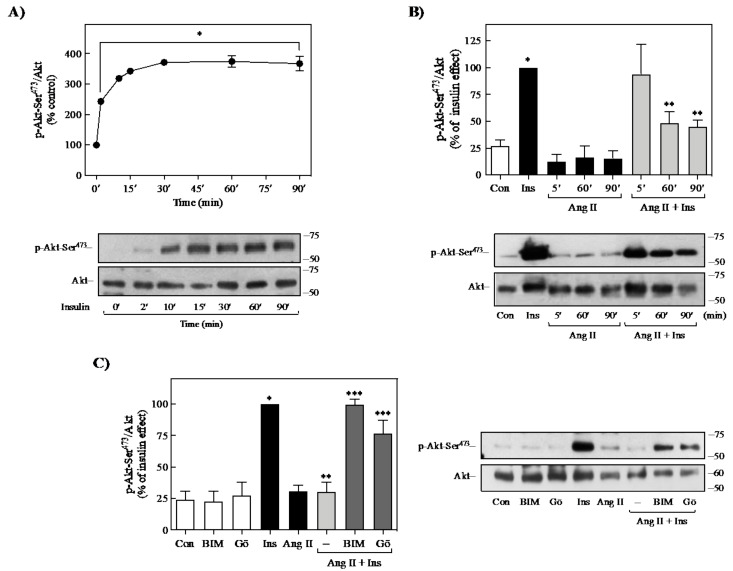
Ang II induces insulin resistance in isolated adipocytes from BALB/c mice. (**A**) Isolated adipocytes were stimulated with 100 nM insulin for the indicated times. (**B**) Cells were pretreated with 100 nM Ang II for the indicated times and then stimulated with 100 nM Ins for 10 min. (**C**) Cells were incubated with 1 μM BIM or 100 nM Gö6976 for 30 min and then stimulated with 100 nM Ang II for 5, 60, or 90 min, and then stimulated with 100 nM insulin for 10 min. Under all experimental conditions, adipose cells without treatment were considered as controls. Total cell lysates were separated by SDS-PAGE and analyzed by immunoblotting with anti-p-Akt Ser^473^, as described in materials and methods. Akt phosphorylation was quantitated by densitometry, and the mean values were plotted from three independent experiments. Vertical lines represent the S.E.M. Representative immunoblots are presented. Western blots were also probed for total Akt as a loading control. (**A**) * *p* < 0.05 vs. time 0′. (**B**) * *p* < 0.05 vs. Con; ** *p* < 0.05 vs. Ins. (**C**) * *p* < 0.05 vs. Con; ** *p* < 0.05 vs. Ins; *** *p* < 0.05 vs. Ang + Ins (--). BIM, BIM I; Con, control; Gö, Gö6976; Ins, insulin.

Further, it was determined if the observed effects of Ang II on insulin signaling in 3T3-L1 adipocytes are reproduced in epididymal isolated adipocytes from BALB/c mice. As shown in Figure 7A, 100 nM insulin induced Akt phosphorylation in a time-dependent manner, reaching a maximum effect at 30 min that continued until 90 min of stimulation. Consistent with the above results, pretreatment of isolated cells with 100 nM Ang II for 60 or 90 min reduced the insulin-induced Akt phosphorylation at Ser^473^ (~70%) (Figure 7B). Interestingly, pretreatment of cells with BIM I recovered the insulin effect on Akt phosphorylation previously reduced by Ang II (Figure 7C). Furthermore, the use of Gö6976, a selective inhibitor of PKCα and PKCβ isoforms [52], partially recovered the inhibitory effect of Ang II on insulin-induced Akt activation. Thus, these results confirm that Ang II can promote insulin signaling desensitization through a mechanism that involves PKC activation in isolated adipocytes.

## 3. Discussion

Classically, the renin-angiotensin system (RAS) is considered the principal mediator of blood pressure, fluid, and electrolyte balance, and its role in metabolic functions has also been demonstrated. In adipose tissue, where a local RAS has been identified, it exerts essential auto/paracrine functions by controlling lipogenesis, lipolysis, and adipogenesis [53,54]. Moreover, RAS has been associated with the pathogenesis of metabolic disorders such as obesity, metabolic syndrome, and insulin resistance, playing a significant role in adipose tissue [53,55]. Interestingly, Ang II, the primary effector hormone of RAS, is implicated in the regulation of insulin signaling in the cardiovascular system, skeletal muscle, and liver, where Ang II, through AT_1_R activation, promotes insulin resistance modulating IRS-1 functions by phosphorylation of specific Ser/Thr residues [4]. However, the exact role of Ang II/AT_1_R in insulin resistance in adipose cells remains controversial. In the present study, we employed the 3T3-L1 adipocyte model to investigate the effect of Ang II on insulin signaling, focusing on the activation of critical proteins involved in insulin signaling. Our results show that exposure of 3T3-L1 adipocytes to Ang II resulted in the inhibition of insulin-stimulated IR and IRS-1-Tyr-phosphorylation, Akt activation, and glucose uptake. These effects were dependent on AT_1_R activation by a mechanism involving PKC-mediated IR-Ser-phosphorylation.

The 3T3-L1 cells used in our study are derived from the original 3T3-Swiss albino mouse fibroblast cell line, and after full differentiation, they express an adipocyte-like phenotype (Supplementary Figure S3). This in vitro cell model of adipocytes expresses endogenous AT_1_ and insulin receptors and helps to study insulin pathways associated with differentiation, metabolism, obesity, insulin resistance, and metabolic diseases [38,56,57]. The above was corroborated in our study since insulin stimulation caused rapid activation of its receptor and downstream substrate IRS-1 to promote Akt phosphorylation and glucose uptake. On the other hand, several groups have previously described the actions of Ang II through the activation of the AT_1_R in 3T3-L1 adipocytes [56,58]. 

In the present study, we found that stimulation of adipocytes with Ang II resulted in the inhibition of insulin-stimulated IR phosphorylation at Tyr^1158^. The Tyr^1158^-residue is included, together with Tyr^1162^ and Tyr^1163^, in the catalytic loop within the tyrosine kinase domains of the receptor. Phosphorylation of these three Tyr-residues is required for full kinase activity of the receptor [3], which subsequently triggers the recruitment and phosphorylation/activation of several endogenous IR substrates, including IRS proteins [5].

We also observed impairment of IRS-1 phosphorylation at Tyr^628^ induced by Ang II. This residue, together with Tyr^608^ (Tyr^612^ in human sequence), is localized in the IRS-1-YXXM motifs, which play an essential role in engaging the p85-regulatory subunit of PI3K. Consequently, decreased phosphorylation at these sites results in defective activation of the PI3K/Akt signaling pathway [45]. Similarly, Andreozzi et al. [45] showed in HUVECs that Ang II inhibits the insulin-stimulated production of nitric oxide through a mechanism associated with the impairment of IRS-1-Tyr^608^/Tyr^628^-phosphorylation. Thus, alteration of IRS-1-Tyr^628^-phosphorylation could explain our results that show reduced insulin-induced phosphorylation of Akt at Ser^473^ and Thr^308^ under the treatment of 3T3-L1 adipocytes with Ang II.

It is well documented that insulin-induced PI3K/Akt signaling pathway is associated with GLUT4 translocation to the cell membrane to stimulate glucose uptake in several cell types, including cardiomyocytes, smooth muscle cells, skeletal muscle cells, and adipocytes [59]. As a consequence of insulin resistance, this cellular response is inhibited by two main mechanisms: decreasing both Akt activity by altering the phosphorylation state of the Ser/Thr-residues associated with the full kinase activity (Ser^473^/Thr^308^) and GLUT4 expression. In agreement with this, we observed that when 3T3-L1 adipocytes were exposed to Ang II, the insulin-induced phosphorylation of Akt at Ser^473^ and Thr^308^ was impaired, an event that affected glucose uptake in these cells. This effect was prevented by the AT_1_R antagonist DuP 753, demonstrating the Ang II/AT_1_R signaling role promoting insulin resistance in adipose cells. Our results are different from those reported by Juan et al. [33], who found that intraperitoneal injection of Ang II to Sprague Dawley rats potentiated insulin-stimulated glucose uptake by adipocytes through AT_1_R activation. In contrast, indirect evidence suggests that through AT_1_R mediated-activation, Ang II decreases insulin sensitivity and promotes insulin resistance in adipose tissue. In this context, Iwai et al. [60] demonstrated that by using the selective AT_1_R blockers, TAK-536, and candesartan, glucose uptake in the adipose tissue from diabetic mice was improved.

Akt also negatively regulate the transcriptional factor forkhead box protein O1 (FoxO1), a critical regulator of metabolism in several cell types and tissues, promoting its phosphorylation at Ser^256^ and nuclear exclusion [61,62,63,64,65]. This inhibition leads to decreased glucose production and increased glucose utilization through glycolysis, glycogen synthesis, and de novo lipogenesis [66]. Chakrabarti and Kandror [41] demonstrated that in 3T3-L1 adipocytes, insulin controls nucleo-cytoplasmic shuttling of FoxO1 and regulates the expression of the rate-limiting lipolytic enzyme adipose triglyceride lipase (ATGL). Moreover, knockdown of FoxO1 in 3T3-L1 adipocytes decreased the expression of ATGL and attenuated basal and isoproterenol-stimulated lipolysis [41,63]. In this context, we found that Ang II reversed the insulin-stimulated phosphorylation of FoxO1 at Ser^256^ in a time-dependent manner, as reported earlier [67], suggesting that Ang II impaired insulin-induced FoxO1 regulation and could participate in the alterations of adipose cells lipid metabolism.

On the other hand, it has been found that IR/IRS-1-Ser/Thr-phosphorylation represents a critical factor promoting the development of insulin resistance and accelerates their degradation [5,6,68]. To explore the molecular mechanisms involved in the Ang II-mediated desensitization of IR signaling observed in 3T3-L1 adipose cells, we decided to evaluate whether Ang II could induce Ser-phosphorylation in both proteins as a regulating mechanism of insulin signaling. Unexpectedly, we found that Ang II induced only Ser-phosphorylation at the IR level without affecting IRS-1-Ser-phosphorylation (Figure 3B,C). This finding is remarkable since most reports have pointed out a significant role of IRS-1 phosphorylation on Ser residues induced by Ang II to impair insulin signaling [4]. Interestingly, Folli et al. [13] reported that pretreatment of rat aortic smooth muscle cells with Ang II increased Ser-phosphorylation of both IR and IRS-1, but unlike our results, they found that Ang II pretreatment affected only Tyr-phosphorylation of IRS by 50%.

In a more general context, previous studies in rats have reported that a high-fat diet (HFD) causes a marked alteration in insulin sensitivity. At the molecular level, this effect was associated with a decrease in insulin-induced IR-Tyr-phosphorylation, an increase in IR-Ser-phosphorylation, and an IR/protein tyrosine phosphatase 1B (PTP1B) association in skeletal muscle and liver tissues [69].

Since PKC represents one of the main kinase effectors of the Ang II/AT_1_R signaling, we decided to evaluate the role of PKC in the actions of Ang II on insulin signaling in 3T3-L1 adipocytes. Two key findings suggested that PKC could be the main Ang II-activated kinase associated with IR-Ser-phosphorylation in 3T3-L1 adipocytes cells. First, using BIM I, a highly selective, cell-permeable, and reversible PKC inhibitor (Ki = 14 nM), we could inhibit the Ang II-induced IR-Ser-phosphorylation (Figure 3D). BIM I acts as a competitive inhibitor for the ATP binding site of PKC and shows high selectivity for PKCα-, β1-, β2-, γ-, δ-, and ε-isozymes [70]. Second, Ang II caused specific PKC-mediated phosphorylation of the IR, as indicated by the phospho-PKC substrate antibody results, that allowed us to detect the PKC-dependent phosphorylation of endogenous IR proteins (Figure 3E). Consistently with these results, we also found that PKC inhibition caused a significant recovery of the Ang II-induced inhibitory effect on insulin-induced IR and Akt activation (Figure 4A,B) and glucose uptake (Figure 4C). Altogether, our results suggest that Ang II/AT_1_R-mediated signaling through PKC activation regulates insulin signaling by impairing IR activity in 3T3-L1 adipocytes.

In vitro assays have shown that PKC promotes phosphorylation of the IR at several Ser/Thr residues located in the juxtamembrane [71], catalytic [72], and carboxyl-terminal regions [73], although to date is not clear what is the regulatory role of each of these sites. In this context, a recent study by Petersen et al. [6] brings to light new evidence about the role of PKC-mediated phosphorylation of the IR. They found that phosphorylation at Thr^1160^ through PKCε mediates hepatic insulin resistance in mice with an HFD. Interestingly, IR-Thr^1160^-phosphorylation would impair IR kinase activity by destabilizing its active conformation. Moreover, in vivo phosphorylation at Ser^994^ promoted by PKC isoforms was detected in IRs isolated from the liver of obese (*fa/fa*) Zucker rats; it was also demonstrated that specific PKC isoforms (α, βI and ζ) could promote the in vitro phosphorylation of the IR on Ser^994^ [72]. In this regard, a more detailed study is required to identify the specific Ser/Thr residues phosphorylated on the IR through Ang II-induced PKC activation.

Previous studies have demonstrated that 3T3-L1 adipocytes express the classical PKC isoenzymes α, β, γ, and the novel PKC isoforms δ and ε [47,74]. Interestingly, and according to these results, we found that Ang II stimulated the activation of classical (α, βI, and βII isoforms) and novel (δ) of PKC isoforms (Figure 5). A relevant finding was that Ang II was able to promote interaction of the IR with the classical PKC isoforms α, βI, and βII, observing the higher interaction with the βII-isoform (~400%, compared with basal interaction) (Figure 6). Interestingly, previous reports have suggested a relevant role of PKCβII in the regulation of the IR activity. Pillay et al. [74] demonstrated that high glucose conditions stimulated the interaction between the PKC isoforms βI, βII, and δ with the IR. However, they found that only the PKCβII interacts with the catalytic domain of the IR, affecting its tyrosine kinase activity. Similarly, it has been shown that Ang II stimulation or the activation of PKCβII can induce phosphorylation of IRS2 in Ser and inhibit its Tyr-phosphorylation on endothelial cells [75].

Another intriguing question is whether the mechanism of IR-Ser-phosphorylation is associated with dephosphorylation/inactivation of the receptor since we could not assume that degradation is implicated in this process. One possibility is the involvement of PTPs in this process. In this context, PTP1B has been associated with IR and IRS-1 Tyr-dephosphorylation as a regulatory mechanism of insulin signaling in 3T3-L1 adipocytes [76]. Based on the present results, we can hypothesize that IR-Ser-phosphorylation could be the required mechanism to promote dephosphorylation of IR by PTP1B and thus desensitize the IR-mediated signaling.

Although the present results clearly show that Ang II induces insulin desensitization in cultured adipocytes, results obtained in isolated adipose cells have been controversial. For example, Ang II was shown to induce insulin signal sensitization [33], while on the other hand, a significant decrease in insulin-induced phosphorylation of Akt, GSK-3, and AS160 by Ang II [35] was also reported in isolated rat adipocytes. Even though these last results are comparable to those presented here, the authors do not demonstrate the mechanism by which Ang II promotes insulin resistance in this tissue. Therefore, we also employed isolated adipocytes from BALB/c mice to determine if similar Ang II effects to those observed in 3T3-L1 adipocytes on insulin signaling can be present in this ex vivo cell model. In this context, we observed that Ang II also affected Akt activation induced by insulin, although the effect was observed after 60 min of pre-stimulation with Ang II.

Similarly, we found that BIM I fully recovered the inhibitory effect of Ang II, suggesting the participation of classical and new PKC isoforms. Furthermore, the use of Gö6976, a selective inhibitor of PKCα and PKCβ isoforms, partially recovered the inhibitory effect of Ang II on insulin-induced Akt activation, which suggests that at least α and β isoforms are involved in this effect. However, a complete analysis is required to figure out the PKC isoforms involved and the molecular target level of regulation.

Our findings broaden the current literature on the molecular mechanisms associated with Ang II-induced insulin resistance in adipose cells, focusing on those from VAT. VAT can produce adipokines and cytokines in excess that lead to the development of a proinflammatory state and insulin resistance, critical players in diseases such as obesity, cardiovascular disorders, and DM2 [77], all of them associated with high levels of Ang II [78]. Although 3T3-L1 adipocytes do not faithfully emulate all the features and functions of adipose cells in vivo, they represent a successful, convenient, and reproducible cell model to study different aspects of adipose cell biology [79,80]. Undoubtedly, 3T3-L1 adipocytes have been widely used to study insulin resistance under controlled conditions, allowing a general understanding of the molecular mechanisms involved in this process [81]. Moreover, differentiated 3T3-L1 adipocytes constitute a highly like model of visceral adipose tissue since they express similar endocrine adipocytic behavior and a local RAS with similar characteristics and functions [78]. For instance, VAT expresses elevated mRNA and protein levels of angiotensinogen (AGT), AT_1_R, and angiotensin converter enzyme (ACE) [82], which can be highly relevant in the mechanism of Ang II-mediated insulin inhibition that we identified in 3T3-L1 cells [78,82].

Considering that epididymal adipose tissue is included in the VAT composition and expresses all the RAS components [77,83,84], our data with isolated adipocytes from epididymal fat (Figure 7) clearly show that Ang II also exerts a desensitizing effect on the insulin signal at the Akt level, an effect dependent on PKC activation. Furthermore, an association has been established between the increase in visceral fat mass and the adipocytes size with the development of hepatic insulin resistance and systemic resistance [85,86,87]. Interestingly, the same relationship has been observed in previous research, in which the co-culture of primary hepatocytes and 3T3-L1 cells showed that insulin resistance in the liver is mediated by adipokines secreted by adipose cells [88,89].

Previous studies have demonstrated that HFD-induced obesity increased plasma Ang II concentrations about 7-fold compared to animals fed a low-fat diet, leading to increased systolic blood pressure [90]. In the same line of evidence, adipocyte AGT deficiency prevented high fat-induced elevations in plasma Ang II concentrations and systolic blood pressure, suggesting that adipose tissue is a major source of Ang II in developing obesity, hypertension, and insulin resistance [90,91]. Additionally, it has been demonstrated that Ang II-derived from VAT in obese subjects with DM2 may regulate adipocyte-derived factors (adipokines) that promote a low-grade inflammatory state and insulin resistance [91].

The association between obesity and insulin resistance has been extensively studied, but there is little evidence of insulin-sensitive obesity, representing a smaller percentage of the obese population [92]. Research in this regard has focused on the relationship between the degree of adiposity and a greater or lesser probability of developing insulin resistance; however, there is evidence of subjects with extreme obesity who retain insulin sensitivity [92,93]. Although Ang II levels in obesity without insulin resistance have not been studied, it has been observed that inhibition of AT_1_Rs with candesartan to prevent hypertension in rats improves adiponectin expression [94]. This is relevant since late-onset obese rats without insulin resistance are characterized by elevated plasma adiponectin levels [95]. These findings suggest a positive relationship between adiponectin and insulin sensitivity, reported in in vitro models of resistance and also studied in patients with metabolic syndrome and diabetes [96,97]. Similar observations have been reported in cohort studies where relevant biochemical parameters have been monitored for the study of the development of insulin resistance and diabetes in obese patients, finding that patients with insulin-sensitive obesity have higher levels of adiponectin compared to those with resistance, in addition to patients without obesity, but with insulin resistance have low levels of adiponectin [92]. This allows us to suggest that in insulin-sensitive obesity conditions, Ang II levels are lower than in the resistance condition.

**Figure 8:**
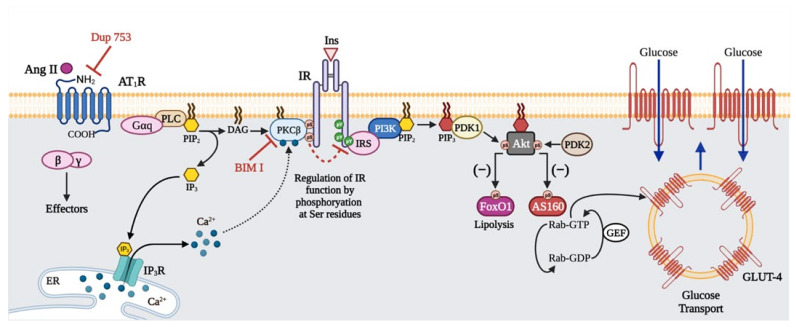
A model of desensitization of the insulin signaling pathway by angiotensin II (Ang II) in 3T3-L1 adipocytes. When adipose cells are stimulated with Ang II, the endogenous AT_1_R is activated, leading to PKCβ activation, which in turn interacts with the insulin receptor (IR) and phosphorylates it on serine (Ser) residues impairing insulin actions such as glucose uptake and regulation of lipolysis. Blue arrows indicate Ang II signaling pathways. Black arrows indicate insulin signaling pathways. Minus sign (−) means negative regulation of FoxO1 and AS160 pathways by Akt. Red lines indicate the effect of PKC inhibitors (BIM I and Gö6976) and AT_1_R antagonist (DuP 753). Red dashed arrow indicates that PKC-mediated Ser-phosphorylation regulates IR function. Figure created with BioRender.com.

In summary, the present findings indicate that in 3T3-L1 differentiated adipocytes Ang II impairs insulin-stimulated IR- and IRS-Tyr-phosphorylation, Akt activation, and glucose uptake by increasing IR-Ser-phosphorylation, by a mechanism dependent on PKC activation (Figure 8). These findings provide new insights that contribute to defining the role of Ang II in developing insulin resistance in adipose cells.

## 4. Materials and Methods

### 4.1. Reagents, Peptides, Inhibitors, and Antibodies

Dulbecco’s modified Eagle’s media (DMEM), fetal bovine serum (FBS), trypsin, antibiotic solutions, and insulin were from Invitrogen/GIBCO (Carlsbad, CA, USA). Bovine calf serum (BCS) was from Hyclone (Logan, UT, USA). Ang II was from BACHEM (Torrance, CA, USA). Bisindolylmaleimide I (BIM I), Gö6976, and protease inhibitor cocktail set III were from Calbiochem (La Jolla, CA, USA). Deoxy-D-glucose,2-[1,2-^3^H(N)] was from PerkinElmer Life and Analytical Sciences (Boston, MA, USA). Bovine and human insulin, 3-isobutyl-1-methylxanthine, dexamethasone, rosiglitazone, cytochalasin B, and PD123177 were from Sigma (St. Louis, MO, USA). Losartan (DuP 753) was a generous gift from DuPont (Wilmington, DE, USA). Immobilon Western Chemiluminescence HRP substrate was from Millipore Corporation (Billerica, MA, USA). Polyclonal and monoclonal primary and secondary antibodies used in the present study are listed in Supplementary Table S1.

### 4.2. Animals

The use of animals in the present study was carried out under the ethical guidelines of the Mexican Official Norm (NOM-062-ZOO-1999) and the National Institutes of Health Guide for the Care and Use of Laboratory Animals (NIH publication updated in 2011). The Institutional Bioethical Committee for Care and Handling of Laboratory Animals at Cinvestav-IPN approved the conducted protocol (No. 0282-18).

### 4.3. Experiments in Isolated Adipocytes

Male BALB/c mice (6 to 8 weeks old; 19.2 ± 1.9 g of body weight) were sacrificed by lethal intraperitoneal sodium pentobarbital injection (>120 mg/kg). The abdominal cavity was opened, and epididymal adipose tissue was removed. Adipose tissue from three mice was used for each experiment. After extraction, epididymal adipose tissue was placed in cold PBS-glucose (5 mM). The tissue was cut into small pieces using forceps and scalpel and placed in digestion solution (PBS-glucose, 0.6 mg of type II collagenase (Sigma) per ml, 50 μM CaCl_2_, 10 mg/mL of BSA) at 37 °C under gentle shaking for 1 h. The digested tissue was filtered and placed in a new 50 mL tube with 15 mL of cold serum-free DMEM: F12 medium (Invitrogen/GIBCO). The isolate was centrifuged for 10 min at 180× *g*, and the layer of adipocytes floating on the surface was taken with trimmed tips and placed in a new 50 mL tube with 15 mL of medium. The isolate was centrifuged for 5 min at 180× *g*. The adipocytes were placed in serum-free DMEM: F12 medium and distributed in 50 mL tubes where the stimuli were performed. At the end of the incubations, the medium was carefully removed with a Pasteur pipette, and Laemmli buffer was added to lyse the cells and prepare the samples for Western Blot [98].

### 4.4. Cell Culture and Differentiation of 3T3-L1 Adipocytes

3T3-L1 cells (American Type Culture Collection, Manassas, VA, USA) were cultured in DMEM-high glucose supplemented with 10% (*v*/*v*) BCS, 100 units/mL penicillin, 100 mg/mL streptomycin (Invitrogen/GIBCO) at 37 °C in 95% air/5% CO_2_. Differentiation protocol of 3T3-L1 cells into adipocytes was conducted, as previously described by Zebisch et al. [99]. Briefly, two days after the cells became confluent, adipocyte differentiation was induced by incubation with Differentiation Medium I (DMI, DMEM-high glucose, and 10% FBS, 1 μg/mL human insulin, 0.25 μM dexamethasone, 0.5 mM 3-isobutyl-1-methylxanthine, 2 μM rosiglitazone). Differentiation Medium II (DMII, DMEM-high glucose containing 10% FBS and 1 μg/mL insulin) was used as a culture medium for two additional days on day two of differentiation. On day four of differentiation, the medium was then changed to DMEM-high glucose with 10% FBS only, and after that, the medium was refreshed every two days for the following two–four days.

### 4.5. Western Blotting

Six hours before each experiment, 3T3-L1-differentiated adipocytes were changed to serum-free DMEM and then treated with the indicated ligand and inhibitors. After treatment, cells were placed on ice, the media was aspirated, and the cells were washed twice with ice-cold PBS and lysed with Laemmli sample buffer 1X. The samples were briefly sonicated, heated at 99 °C, and centrifuged for 5 min at 13,000 rpm. The supernatant was electrophoresed on SDS-PAGE (8 or 10%) and transferred to PVDF membranes. Then, membranes were incubated overnight at 4 °C with primary antibodies.

To detect the presence of phosphorylated sites on Ser/Thr residues by PKC in the IR, we employed the phospho-PKC substrate antibody (Cell Signaling Technology, Danvers, MA, USA) that detects proteins containing phospho-Ser/Thr residues in the motif (R/K)X(pS)(Hyd)(R/K), where Hyd is any hydrophobic amino acid, and X is any amino acid. Other primary antibodies used in the present study are described in Supplementary Table S1. After incubation with primary antibodies, membranes were washed three times with TBST before probing with horseradish peroxidase-conjugated secondary antibodies for 1h at room temperature. Blots were then visualized with chemiluminescence reagent. Autoradiograms were scanned using GS-800 Calibrated Imaging Densitometer (Bio-Rad, Hercules, CA, USA), and the labeled bands were quantified using Quantity One 4.6.3 software program (Bio-Rad).

### 4.6. Glucose Uptake Assay

The effect of insulin and Ang II on glucose uptake in 3T3-L1 adipocytes was determined by measuring [^3^H]-2-deoxy-glucose (2-DG) uptake, as previously described, with some changes [100]. 3T3-L1 adipocytes were washed once with KRH saline solution (118 mM NaCl, 2.4 mM KCl, 0.8 mM MgCl_2_, 1.8 mM CaCl_2_, 20 mM HEPES, 2% BSA, pH 7.4) and then incubated in the same solution with insulin or Ang II, for the indicated times and concentrations in the presence or absence of inhibitors at 37 °C. After incubation, cells were rinsed twice with HEPES-buffered saline, and glucose uptake was quantitated by exposing the cells to 1 μCi/mL [^3^H]-2-DG for 15 min at room temperature. At the end of the 15 min period, the supernatant was aspirated rapidly, and the cells were washed twice with ice-cold KRH buffer. The cells were lysed in 0.1% SDS, and the associated radioactivity was determined by liquid scintillation counting (Beckman LS6000TA) and normalized according to the total protein level. Nonspecific uptake was determined in the presence of 20 µM cytochalasin B and subtracted from all values. Each experiment was assayed by duplicate.

### 4.7. Immunoprecipitation Assay for Serine Phosphorylated IR and IRS

Differentiated 3T3-L1 adipocytes were grown in 10-cm dishes and serum-starved for 12 h before treatment with agonists (100 nM Ang II or 100 nM insulin) for the indicated times at 37 °C. Cells were washed twice with ice-cold DPBS and lysed in Nonidet P-40 solubilization buffer (25 mM NaCl, 50 mM HEPES, 0.5% Nonidet P-40, 10% glycerol, 2 mM EDTA, pH 8.0, containing protease and phosphatase inhibitors). After immunoprecipitation of IR or PKC with protein A/G PLUS-Agarose (Santa Cruz Biotechnology, Santa Cruz, CA, USA), the proteins were resolved by SDS-PAGE, Western-blotted, and probed with anti-phospho-Ser, followed by a horseradish peroxidase conjugate to identify phosphorylated proteins. Blots were visualized and quantified as described.

### 4.8. Co-Immunoprecipitation Assay

Differentiated 3T3-L1 adipocytes were grown in 10-cm dishes and serum-deprived for 12 h before treatment with 100 nM Ang II for 60 min or pretreatment with 10 µM DuP for 30 min and treatment with 100 nM Ang II for an additional 60 min at 37 °C. Under all experimental conditions, cells that were not treated were considered as controls. Cells were washed twice with ice-cold PBS and lysed in Nonidet P-40 solubilization buffer (50 mM Tris-HCl, 150 mM NaCl, 2 mM Orthovanadate, 1 mM NaF, 1% Nonidet P-40, 10% Glycerol, 2 mM EDTA, pH 7.4, containing protease inhibitors). After immunoprecipitation of insulin receptors with anti-IR-β polyclonal antibody (Santa Cruz Biotechnology) and protein A/G PLUS-Agarose (Santa Cruz Biotechnology), the proteins were resolved by SDS-PAGE, Western blotted, and probed with anti-PKCα, -PKCβI, -PKCβII, or -PKCδ polyclonal antibodies (Santa Cruz Biotechnology), followed by a horseradish peroxidase conjugate to identify co-immunoprecipitated proteins. Blots were also stripped with stripping buffer (100 nM Glycine-HCl, pH 2.7) and reprobed with anti-IR-β polyclonal antibody (Santa Cruz Biotechnology). Blots were visualized and quantified, as indicated above.

### 4.9. Statistical Analysis

Measurements of intensity from Western blots were analyzed using either one- or two-way ANOVA with Dunnet and Bonferroni’s post-test using PRISM, version 8.0b (GraphPad Software, San Diego, CA, USA). In all cases, *p* < 0.05 was considered to be significant. Resultant data were plotted, with data expressed as mean ± S.E.M. from at least three independent experiments, and representative blots are shown.

## Data Availability

The data presented in this study are available on request from the corresponding author.

## Supplementary Materials

The following supporting information can be found at: https://www.mdpi.com/article/10.3390/ijms23116048/s1.

## Abbreviations

Ang II	Angiotensin II
AGT	Angiotensinogen
AT_1_R	Angiotensin receptor type 1
AT_2_R	Angiotensin receptor type 2
ATGL	Adipose triglyceride lipase
BIM I	Bisindolylmaleimide I
DM2	Type 2 diabetes mellitus
FoxO1	Forkhead box protein O1
GLUT4	Glucose transporter 4
GSK-3	Glycogen synthase kinase-3
HFD	High-fat diet
IR	Insulin receptor
IRS	Insulin receptor substrate
PI3K	Phosphatidylinositol 3-kinase
PKC	Protein kinase C
PTPs	Protein tyrosine phosphatases
PTP1B	Protein tyrosine phosphatase 1B
RAS	Renin-angiotensin system
ROS	Reactive oxygen species
Ser	Serine
SOCS	Suppressor of cytokine signaling
Thr	Threonine
Tyr	Tyrosine
VAT	Visceral adipose tissue
