# Supplementary material for: Angiotensin II Inhibits Insulin Receptor Signaling in Adipose Cells
Source: Int J Mol Sci. 2022 May 27;23(11):6048. doi: 10.3390/ijms23116048 (PMC9181642; doi:10.3390/ijms23116048)
Supplement: Supplementary file 1 [file ijms-23-06048-s001.zip › ijms-1662471-supplementary.pdf]

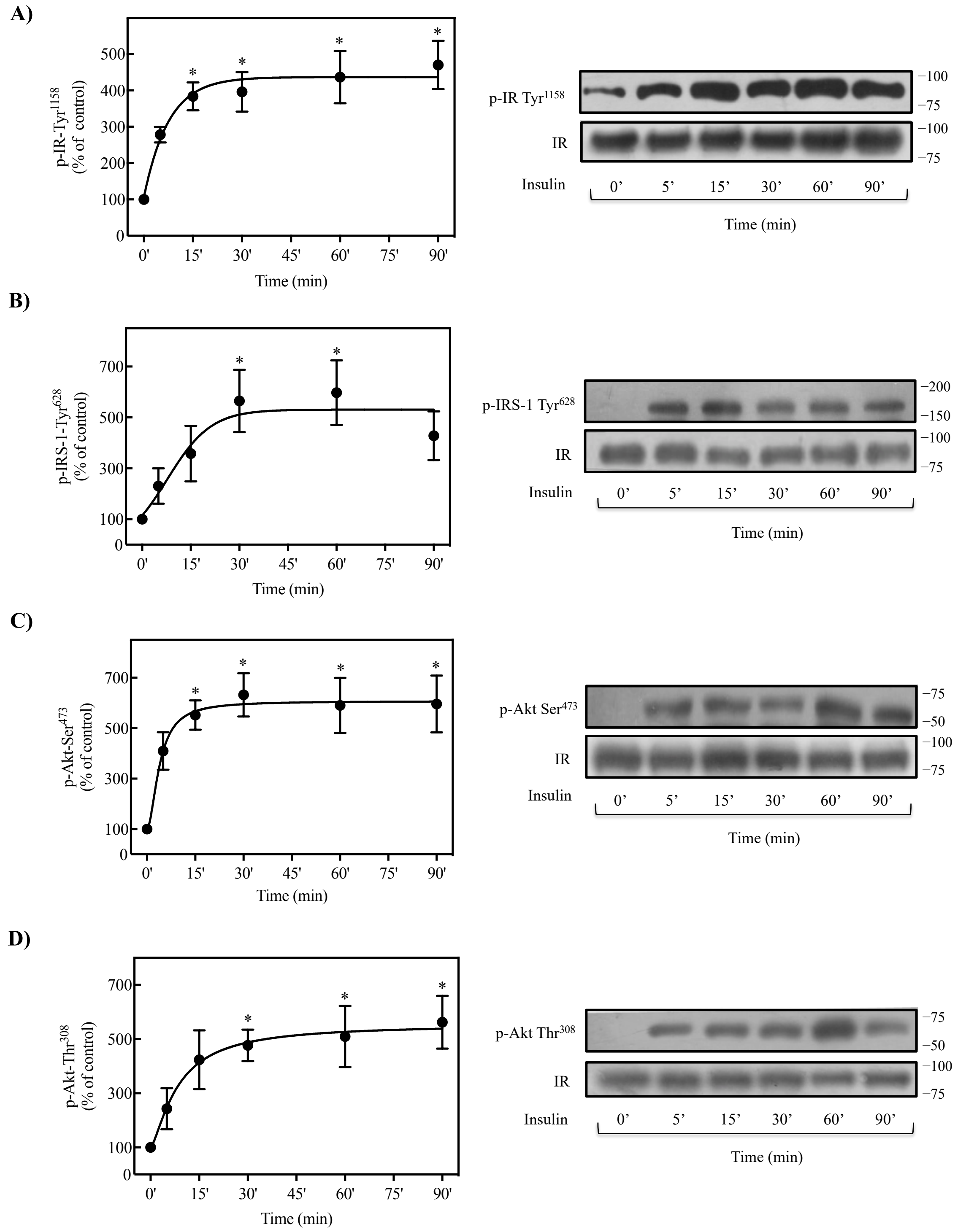

Supplementary Figure S1. ***Effect of insulin on IR, IRS, and Akt phosphorylation.*** 3T3-L1 adipocytes were treated with 100 nM insulin for the indicated times. Total cell lysates were separated by SDS-PAGE and analyzed by immunoblotting with anti-p-IR Tyr^{1158} **(A)**, anti-p-IRS Tyr^{628} **(B)**, anti-p-Akt Ser^{473} **(C)** or anti-p-Akt Thr^{308} **(D)**, as described in materials and methods. IR, IRS, and Akt phosphorylation was quantitated by densitometry, and the mean values were plotted from three-five independent experiments. Vertical lines represent the S.E.M. Representative immunoblots are presented. Western blots were also probed for total IR, as loading control. $^{*}p < 0.05$ *vs* time 0' (control).

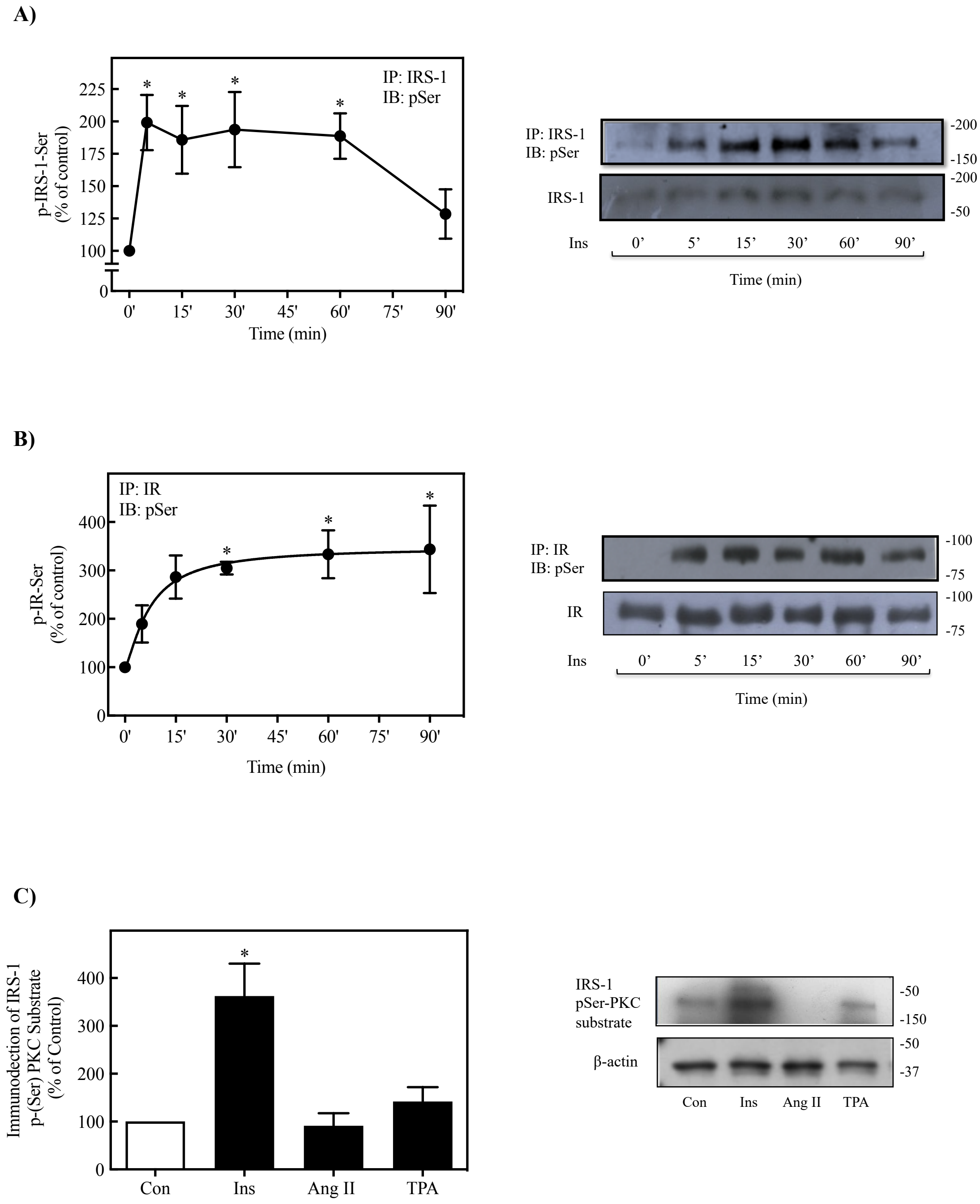

Supplementary Figure S2. ***Insulin promotes IR- and IRS-1-Ser-phosphorylation***. **(A and B)** 3T3-L1 adipocytes were stimulated with 100 nM insulin for the indicated times. Total cell lysates were immunoprecipitated with anti-IRS-1 **(A)** or anti-IR **(B)** antibody before SDS-PAGE analysis and immunoblotted with an anti-phospho-Ser antibody. **(C)** 3T3-L1 adipocytes were stimulated with 100 nM insulin, Ang II, or TPA for 60 minutes. Total cell lysates were immunoprecipitated with anti-IRS-1 antibody before SDS-PAGE analysis and immunoblotted with an anti-phospho-(Ser) PKC substrate antibody. Blots were quantitated by densitometry, and the mean values were plotted from three-five independent experiments. Vertical lines represent the S.E.M. Representative immunoblots are presented. Western blots were also probed for total IRS-1 **(A)**, IR **(B)** or β-actin **(C)**, as loading control. **(A and B)** $^{*}p < 0.05$ *vs* time 0’ (control). **(C)** $^{*}p < 0.05$ *vs* control. Con, control; Ins, insulin; TPA, 12-O-Tetradecanoylphorbol-13-acetate

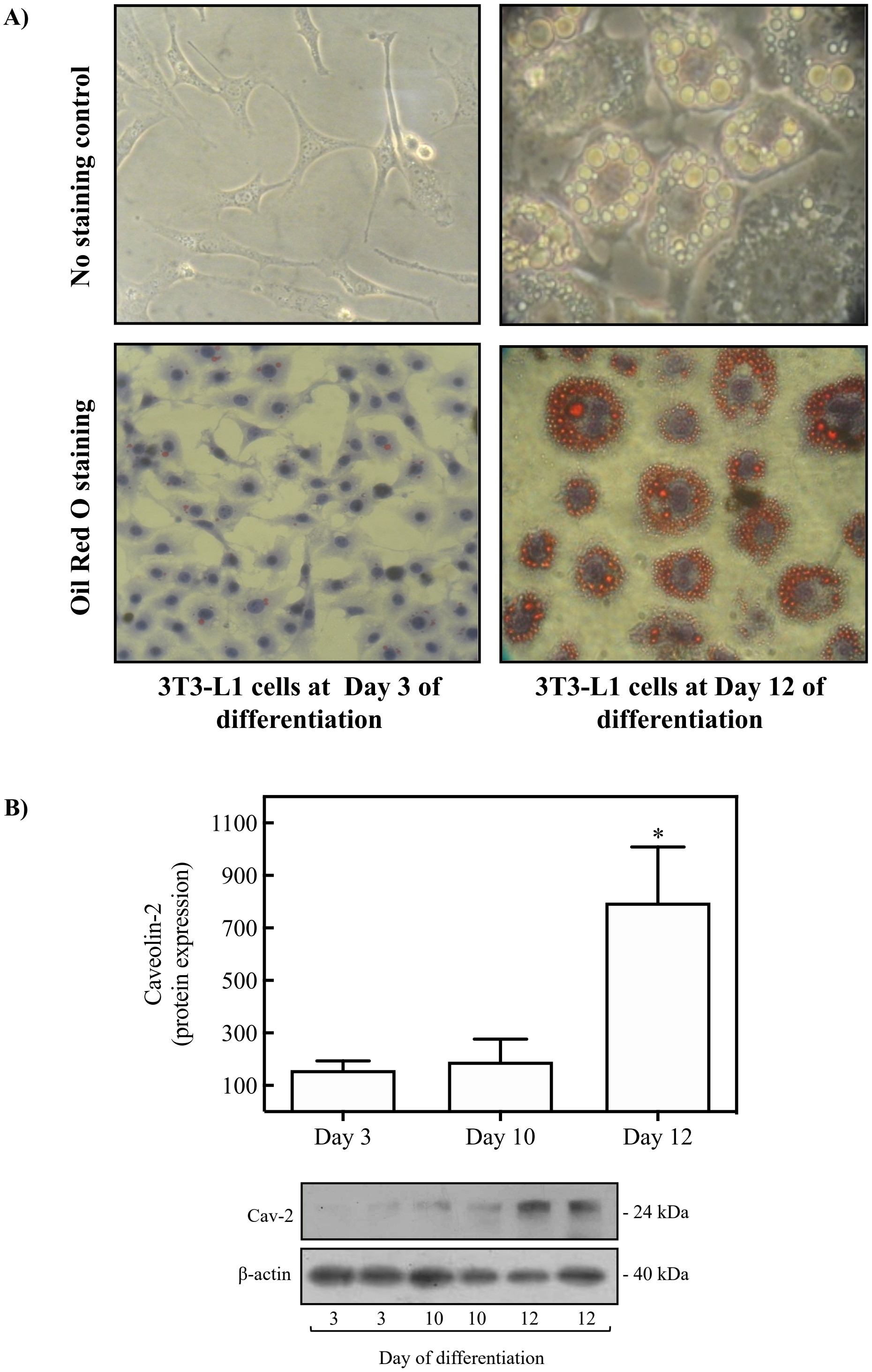

Supplementary Figure S3. ***Differentiation of 3T3-L1 cells into adipocytes*.** **(A)** Lipid accumulation in 3T3L1 adipocytes after 3 and 10 days of induction of adipogenic differentiation. Cells were fixed, and lipid droplets were stained with Oil Red O dye and visualized with an Olympus CKX41 optical microscope. **(B)** 3T3-L1 cells were lysed on days 3, 10, and 12 of the differentiation processes. Total cell lysates were separated by SDS-PAGE and analyzed by immunoblotting with anti-caveolin-2. Blots were quantitated by densitometry, and the mean values from three independent experiments were plotted. *$p < 0.05$ vs days 3 and 10.

Primary antibodies	***Source***	***Catalog no.***	***Clone***	***Dilutions***
p-Akt (Ser^{473})	Santa Cruz	sc-7985	Rabbit polyclonal	1:6000
p-Akt (Thr^{308})	Santa Cruz	sc-135650	Rabbit polyclonal	1:6000
p-IRS-1 (Tyr^{632})	Santa Cruz	sc-17196	Rabbit polyclonal	1:4000
p-PKCα (Ser^{657})	Santa Cruz	sc-12356	Goat polyclonal	1:3000
p-PKCε (Ser^{729})	Santa Cruz	sc-12355	Rabbit polyclonal	1:3000
*IR-β	Santa Cruz	sc-711	Rabbit polyclonal	1:1000
PKCα	Santa Cruz	sc-208	Rabbit polyclonal	1:3000
*PKCβI	Santa Cruz	sc-209	Rabbit polyclonal	1:3000
PKCβII	Santa Cruz	sc-210	Rabbit polyclonal	1:3000
PKCδ	Santa Cruz	sc-213	Rabbit polyclonal	1:3000
PKCε	Santa Cruz	sc-214	Goat polyclonal	1:6000
β-Actin	Santa Cruz	sc-1616	Goat polyclonal	1:5000
p-FKHR (Ser^{256})	Cell Signaling	#9461	Rabbit polyclonal	1:3000
p-IRS-1 (Ser^{612})	Cell Signaling	#2386	Rabbit polyclonal	1:4000
p-PKCα/βII ($Thr^{638/641}$)	Cell Signaling	#9375	Rabbit polyclonal	1:6000
p-PKCδ/θ ($Ser^{643/676}$)	Cell Signaling	#9276	Rabbit polyclonal	1:6000
p-Ser-PKC substrate	Cell Signaling	#2261	Rabbit polyclonal	1:6000
*IRS-1	Cell Signaling	#2382	Rabbit polyclonal	1:1000
p-IR (Tyr^{1158})	Invitrogen	44-802G	Rabbit polyclonal	1:3000
p-IRS-1 (Tyr^{632})	Merck Millipore	#09-433	Mouse monoclonal	1:4000
p-Ser (clone 4A4)	Merck Millipore	#05-1000	Mouse monoclonal	1:2000
Secondary antibodies	***Source***	***Catalog no.***	***Clone***	***Dilutions***
Goat IgG-HRP	Jackson ImmunoResearch	305-035-003	Rabbit polyclonal	1:5000
Mouse IgG-HRP	Santa Cruz	sc-2005	Goat polyclonal	1:5000
Rabbit IgG-HRP	Santa Cruz	sc-2004	Goat polyclonal	1:5000

Supplementary Table S1. ***Table of antibodies used for western blot and *immunoprecipitation studies.***

Supplementary Methods

Oil red O staining in 3T3-L1 cells

Lipid accumulation in 3T3L1 adipocytes was evaluated by Oil Red O staining after 3 and 10 days of adipogenic differentiation induction. After washing with 2 ml of DPBS, cell cultures were fixed for 30 min with 10% formalin. Then, cells were washed with sterile double distilled water and subsequently with 99% isopropanol for 2 min and stained with a filtered Oil Red O solution with 2 parts of deionized water and allow to sit at room temperature for 10 minutes. Cells were washed with sterile double distilled water and stained with Mayer's Hematoxylin Bio-Optica ready to use solution for 1 min at room temperature and washed again with sterile double distilled water (AdipoRed™ Adipogenesis Assay Reagent from LONZA). The cells were observed with an optical microscope Olympus CKX41 20X and Nikon Digital camera.
